# Herpes zoster mimicking sciatica: A diagnostic pitfall in immunocompetent middle-aged adult with low back pain

**DOI:** 10.1016/j.idcr.2026.e02598

**Published:** 2026-05-04

**Authors:** Daisuke Hori, Yuichi Sakamoto

**Affiliations:** a Occupational and Aerospace Psychiatry Group, Institute of Medicine, University of Tsukuba, Tsukuba, Ibaraki, Japan; b Department of Mental Health, Social Psychiatry, and Occupational Psychiatry, University of Tsukuba Hospital, Tsukuba, Ibaraki, Japan

**Keywords:** Herpes zoster, Radiculopathy, Diagnostic error, Cognitive bias

## Abstract

Herpes zoster (HZ) involving lumbosacral dermatomes can mimic sciatica, leading to diagnostic delays. While HZ is more common in older or immunocompromised individuals, it can occur in immunocompetent middle-aged adults, presenting diagnostic challenges due to atypical presentations, particularly in patients with chronic pain. We report a 40-year-old immunocompetent male with chronic low back pain who presented with an erythematous gluteal rash and acute radiating left leg pain following a chiropractic session. The rash was initially misdiagnosed as eczema at a dermatology clinic, and the leg pain was attributed to lumbar radiculopathy due to the temporal association with mechanical intervention. The patient’s own attribution of symptoms, together with the initial clinical framing, led to a delay in recognizing the underlying viral etiology. By Day 6, the clinical evolution of the rash into grouped vesicles and the presence of marked allodynia, despite a negative straight leg raise test, supported the diagnosis of lumbosacral HZ. Treatment with oral famciclovir resulted in rapid symptomatic improvement. This case underscores the importance of considering HZ in the differential diagnosis of acute radicular pain, even in the absence of typical early vesicular eruptions. It also highlights how cognitive biases—such as anchoring to a musculoskeletal cause and framing by the primary dermatological complaint—can impede diagnostic reasoning. Clinicians should maintain a high index of suspicion for HZ in patients with unilateral radicular pain and remain vigilant against cognitive pitfalls to ensure timely antiviral intervention.

## Highlights

• Herpes zoster can mimic sciatica in immunocompetent adults with back pain.
• Pain–rash location discordance complicated clinical recognition.
• Patient’s cognitive bias contributed to misdiagnosis and delay.

A 40-year-old immunocompetent male physician with chronic low back pain developed acute left leg pain radiating along the posterior thigh and a pruritic erythematous lesion in the gluteal cleft. He had a remote history of herpes zoster (HZ) affecting the left lateral abdomen 20 years earlier, during a period of high psychological stress.

**Fig. 1:**
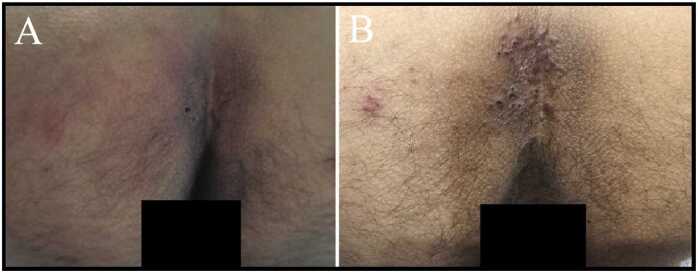
Clinical evolution of herpes zoster involving the gluteal cleft. (A) Appearance on Day 5, one day before the diagnosis. A broad erythematous patch is centered on the intergluteal cleft and extends onto the left buttock. Lesions within the cleft have already progressed to early crusts. (B) Appearance on Day 7, after one day of antiviral therapy. The older, central lesions have evolved into dense, crusted papules involving the midline, while grouped vesicles have become prominent on the erythematous patch on the left buttock.

On Day 1, he underwent routine chiropractic manipulation for chronic low back pain. On Day 2, he developed left-sided posterior thigh pain, sharp and burning in quality, worsening at night and unrelieved by rest or positional changes. On Day 3, he visited a dermatology clinic primarily for an unrelated neck wart. The gluteal lesion was noted incidentally and diagnosed as eczema; a topical corticosteroid was prescribed. The patient did not mention his leg pain, having attributed it to the recent chiropractic session — a classic example of anchoring bias. The rash continued to expand despite corticosteroid application. By Day 5, it appeared as a broad erythematous patch with early crusting in the intergluteal cleft (Fig. 1A). On Day 6, re-evaluation at a second dermatology clinic revealed marked allodynia over the gluteal cleft, a negative straight leg raise test, and no motor deficits or sphincter disturbances. HZ was diagnosed clinically without laboratory confirmation, which reflects a common real-world scenario where early diagnosis must be made without confirmatory testing. Oral famciclovir (500 mg three times daily) and loxoprofen were initiated for 7 days, with rapid improvement in neuropathic pain. By Day 7, crusted papules occupied the intergluteal cleft with grouped vesicles extending onto the left buttock (Fig. 1B).

Multiple interacting factors contributed to the delayed diagnosis. HZ is less commonly suspected in immunocompetent adults under 50 [1]. The pre-vesicular rash in the anatomically concealed gluteal cleft resembled dermatitis. The cutaneous findings were localized to the gluteal cleft (S2 dermatome), while the dominant symptom was posterior thigh pain, creating an apparent dermatomal dissociation that can occur due to overlapping dermatomes and variability in sensory innervation [2], [3]. The temporal association with chiropractic manipulation and a history of chronic back pain reinforced anchoring to a mechanical etiology [4]. The patient's framing of the consultation around an unrelated complaint further diverted clinical attention. This case highlights that HZ should be considered in acute unilateral radicular pain even without vesicular eruptions, and that anchoring, framing, and premature closure can delay antiviral therapy and increase the risk of postherpetic neuralgia [5].

## CRediT authorship contribution statement

**Daisuke Hori:** Writing – review & editing, Writing – original draft, Visualization, Validation, Supervision, Resources, Project administration, Methodology, Investigation, Funding acquisition, Data curation, Conceptualization. **Yuichi Sakamoto:** Writing – review & editing, Visualization, Validation.

## Consent

Written informed consent was obtained from the patient for publication of this case report and accompanying images.

## Ethical approval

Ethical approval is not required for this case report in accordance with local/institutional guidelines.

## Declaration of Generative AI and AI-assisted technologies in the writing process

During manuscript preparation, an AI-assisted tool (Gemini 3 Flash, Google) was used solely for language improvements, grammar corrections, and reference formatting, in accordance with journal policy. The authors take full and sole responsibility for the integrity, accuracy, and originality of the final scientific content and all cited references.

## Funding statement

This work was supported by the Japan Society for the Promotion of Science (10.13039/501100001691JSPS) KAKENHI (Grant no.: JP24K20698). The JSPS had no role in the study design, data collection, analysis, interpretation, or manuscript preparation.

## Conflict of interest

Authors declare no conflict of interests to disclose.

## Declaration of Competing Interest

The authors declare the following financial interests/personal relationships which may be considered as potential competing interests: Daisuke Hori reports financial support was provided by Japan Society for the Promotion of Science. If there are other authors, they declare that they have no known competing financial interests or personal relationships that could have appeared to influence the work reported in this paper.
